# Research on Optimal Policy of Single-Period Inventory Management with Two Suppliers

**DOI:** 10.1155/2014/417319

**Published:** 2014-11-20

**Authors:** Baimei Yang, Lihui Sui, Peipei Zhu

**Affiliations:** School of Business, Shanghai Dianji University, Shanghai 201306, China

## Abstract

We study a single-period inventory control problem with two independent suppliers. With the first supplier, the buyer incurs a high variable cost but negligible fixed cost; with the second supplier, the buyer incurs a lower variable cost but a positive fixed cost. At the same time, the ordering quantity is limited. We develop the optimal inventory control policy when the holding and shortage cost function is convex. We also conduct some numerical experiments to explore the effects of the fixed setup cost *K* and the ordering capacity *Q* on the optimal control policy.

## 1. Introduction

In this paper, we consider a single-period inventory system with two suppliers and different ordering cost structures. We assume that the two suppliers are independent of each other, which indicates that the decision of one supplier will not influence the other one's decision. And we could only order from one supplier. At the same time, the ordering quantity is limited.

Several studies have been conducted attempting to extend the analysis of a single product system with two suppliers. Fox et al. [1] analyze a periodic-review inventory model where the decision maker can buy from either of the two suppliers. With the first supplier, the buyer incurs a high variable cost but negligible fixed cost; with the second supplier, the buyer incurs a lower variable cost but a substantial fixed cost. Consequently, ordering costs are piecewise linear and concave. Chen et al. [2] establish a new preservation property of quasi-*K*-concavity under certain optimization operations and apply the result to analyze joint inventory-pricing models for single-product periodic-review inventory systems with concave ordering costs. Caliskan-Demirag et al. [3] consider a stochastic periodic-review inventory control system in which the fixed cost depends on the order quantity.

This problem is also related to the optimal control of a single-product system with finite capacity and setup cost. When the setup cost is zero, Federgruen and Zipkin [4, 5] have shown that the optimal strategy for the capacitated inventory control problem is known as the modified base-stock policy. Several studies have been conducted on this problem. For instance, Shaoxiang and Lambrecht [6] and Chen [7] point out that the generally known result is that the optimal policy can only be partially characterized in the form of *X*-*Y* bands. In Gallego and Scheller-Wolf [8], the structure of the policy between the bands is further refined using two numbers *s* and *s*′ in four possible regions. Chao et al. [9] have studied a dynamic inventory and pricing optimization problem in a periodic review inventory system with setup cost and finite ordering capacity in each period. Gavish and Graves [10] study one-product production/inventory problem with a fixed setup cost under continuous review policy. De Kok [11] deals with a one-product production/inventory model with lost sales.

If the decisions of the two suppliers can affect each other, this problem is changed into a problem with capacity constraints and game theory. Some experts have made deep studies on this area. Nie [12] considers commitment for storable goods under vertically integrated structures. Nie and Chen [13] and Nie [14] focus on the duopoly substitutability product with an upstream input subjected to capacity constraints. Chen and Wen [15] analyze co-op advertising behavior based on a dual-brand model with a single manufacturer and a single retailer. And more applications of game theory to problems in economics can be found in Nie et al. [16].

In this paper, we consider a single-period inventory system with two independent suppliers and investigate the structure of the optimal inventory control. When we order from the first supplier, the ordering cost only includes variable cost. When we order from the other supplier, the ordering costs include a fixed cost and variable cost. Moreover, the ordering capacity is finite. Then we establish the model and develop the optimal policy for both general convex holding and shortage cost function and piecewise linear holding and shortage cost function. We also conduct several numerical experiments to explore the effects of the fixed ordering cost *K* and the ordering capacity *Q* on the optimal control policy.

The rest of this paper is organized as follows. In the next section we present the model. The main result and the proofs are provided in Section 3. In Section 4 are the numerical results, and concluding remarks are in Section 5.

## 2. Model

Consider a single-period inventory system with two independent suppliers. Here, the decision of one supplier will not influence the other one's decision. When we order from the first supplier, the ordering cost only includes variable cost *c*_1_. When we order from the other supplier, the ordering costs include a fixed cost *K* and variable cost *c*_2_. Here, *c*_1_ > *c*_2_. And we could only order from one supplier.

In the single-period inventory system, the initial inventory level is zero. There is a finite ordering capacity *Q* for both suppliers. That is, the ordering quantity from each supplier cannot exceed *Q*. Let *x* be the order quantity. Then there will be 0 ≤ *x* ≤ *Q*.

The sequence of events during a period is as follows: (1) replenishment order is placed, (2) replenishment order arrives, (3) random demand is realized, and (4) all costs are computed.

In the period, there are *N* nonnegative demands, from *D*_1_ to *D*_*N*_. The possibility for *D*_*n*_ is *p*_*n*_, *n* = 1,…, *N*. And ∑_*n*=1_^*N*^ *p*_*n*_ = 1. A cost *H*(*x*) is incurred at the end of period if the inventory level after demand realization is *x*, which represents inventory holding cost if *x* ≥ 0 and shortage cost if *x* < 0. Assume that *H*(*x*) is convex. Then, the expected holding and shortage cost, given that the inventory level after replenishment is *x*, is *E*[*H*(*x* − *D*_*n*_)], which is also convex.

Let *C*_*i*_ denote the minimum cost when purchasing from supplier *i*. Then,

(1) C1=min⁡0≤x≤Qc1x+EHx−Dn,C2=min⁡0≤x≤QKIx>0+c2x+EHx−Dn,

where 1[*A*] is the indicator function, taking value 1 if statement *A* is true and zero otherwise. The objective is to characterize the optimal ordering strategy that minimizes the expected cost. Hence, the optimal equation is

(2) C=min⁡{C1,C2}=min⁡0≤x≤Qttc1x+E[H(x−Dn)], hhhihhKIx>0+c2x+EHx−Dntt.

## 3. Analysis and Results

In this section, we will analyze the two suppliers separately first. Then we compare the two cost functions and obtain the results. In addition, we will give one special case.

### 3.1. Supplier 1

The cost function of ordering from supplier 1 is simple. Due to the convexity of *E*[*H*(*x* − *D*_*n*_)], it is obvious that *c*_1_ *x* + *E*[*H*(*x* − *D*_*n*_)] is also convex. Hence, it is easy to obtain the optimal policy for ordering from supplier 1.

**Lemma 1 .**

Assume that

(3) $$\begin{array}{c} c_{1}x_{1}+E\left[H\left(x_{1}-D_{n}\right)\right]=\min\left\{c_{1}x+E\left[H\left(x-D_{n}\right)\right]\right\}. \end{array}$$

Then the optimal policy when ordering from supplier  1 is

1. no order if *x*_1_ ≤ 0;
2. order *x*_1_ if 0 < *x*_1_ < *Q*; and
3. order capacity *Q* if *x*_1_ ≥ *Q*.

### 3.2. Supplier 2

The cost function of ordering from supplier 2 is complex. Actually, the cost function could be restructured as

(4) $$\begin{array}{c} C_{2}=\underset{0\leq x\leq Q}{\min}\left\{E\left[H\left(-D_{n}\right)\right],K+c_{2}x+E\left[H\left(x-D_{n}\right)\right]\right\}. \end{array}$$

It is obvious that *c*_2_ *x* + *E*[*H*(*x* − *D*_*n*_)] is also convex. Then we will obtain the following lemma.

**Lemma 2 .**

Assume that

(5) c2x2+EHx2−Dn=min⁡c2x+EHx−Dn,k1=E[H(−Dn)]−c2min⁡{x2,Q}hhhh−E[H(min⁡{x2,Q}−Dn)].

Then the optimal policy when ordering from supplier 2 is

1. no order if *x*_2_ ≤ 0;
2. order *x*_2_ if  0 < *x*_2_ < *Q* and *K* < *k*_1_;
3. no order if  0 < *x*_2_ < *Q* and *K* ≥ *k*_1_;
4. order capacity *Q* if *x*_2_ ≥ *Q* and *K* < *k*_1_; and
5. no order if *x*_2_ ≥ *Q* and *K* ≥ *k*_1_.

From Lemma 2, we could regard *k*_1_ as a benchmarking of *K*. If *K* ≥ *k*_1_, the optimal policy of ordering from supplier 2 is always no order.

### 3.3. Combination

From the previous analysis, we will find that the values of both *x*_1_ and *x*_2_ are independent of ordering capacity *Q* and fixed ordering cost *K*. Moreover, due to *c*_1_ > *c*_2_, there is always *x*_1_ < *x*_2_. Then there would be six possibilities: *x*_1_ < *x*_2_ ≤ 0, *x*_1_ ≤ 0 < *x*_2_ < *Q*, *x*_1_ ≤ 0 < *Q* < *x*_2_, 0 < *x*_1_ < *x*_2_ < *Q*, 0 < *x*_1_ < *Q* < *x*_2_, and *Q* ≤ *x*_1_ < *x*_2_.

The following theorem characterizes the structure of the optimal inventory and pricing policy for each period.

**Theorem 3 .**

Suppose that

(6) c1x1+EHx1−Dn=min⁡c1x+EHx−Dn,c2x2+EHx2−Dn=min⁡c2x+EHx−Dn,k1=E[H(−Dn)]−c2min⁡{x2,Q}hhhh−EHmin⁡x2,Q−Dn,k2=c1min⁡{x1,Q}+E[H(min⁡{x1,Q}−Dn)]hhhh−c2min⁡{x2,Q}−E[H(min⁡{x2,Q}−Dn)].

If *K* ≥ *k*_1_, then the optimal ordering policy is

1. no order if *x*_1_ ≤ 0;
2. order *x*_1_ from supplier 1 if 0 < *x*_1_ < *Q*; and
3. order capacity *Q* from supplier 2 if *x*_1_ ≥ *Q*.

And if *K* < *k*_1_, then the optimal ordering policy is

1. no order if *x*_1_ < *x*_2_ ≤ 0;
2. order *x*_2_ from supplier 2 if *x*_1_ ≤ 0 < *x*_2_ < *Q*;
3. order capacity *Q* from supplier 2 if *x*_1_ ≤ 0 < *Q* < *x*_2_;
4. order *x*_2_  from supplier 2 if  0 < *x*_1_ < *x*_2_ < *Q* and *K* ≤ *k*_2_;
5. order *x*_1_  from supplier 1 if  0 < *x*_1_ < *x*_2_ < *Q* and *K* ≥ *k*_2_;
6. order capacity *Q* from supplier 2 if 0 < *x*_1_ < *Q* < *x*_2_ and *K* ≤ *k*_2_;
7. order *x*_1_ from supplier 1 if   0 < *x*_1_ < *Q* < *x*_2_ and *K* ≥ *k*_2_;
8. order capacity *Q* from supplier 2 if *x*_2_ > *x*_1_ ≥ *Q* and *K* ≤ *k*_2_; and
9. order capacity *Q* from supplier 1 if *x*_2_ > *x*_1_ ≥ *Q* and *K* ≥ *k*_2_.

Here we omit the proof of Theorem 3. From Theorem 3, we will find if *K* ≥ *k*_1_;we will not order from supplier 2. On the contrary, when *K* < *k*_1_,   we will not order from supplier 1 if *x*_1_ < 0. Moreover, when *K* < *k*_1_ and *x*_2_ > *x*_1_ > 0, we will order min⁡{*x*_2_, *Q*} from supplier 2 if *K* ≤ *k*_2_ and min⁡{*x*_1_, *Q*} from supplier 1 if *K* ≥ *k*_2_. If *K* = *k*_2_, ordering from either supplier could be optimal.

### 3.4. Special Case

In the previous analysis, we only assume that *H*(*x*) is convex. The most common function of holding and shortage cost is piecewise linear; for instance, (*x*) = *hx* ^+^ + *bx* ^−^. Here *x* ^+^ = max⁡{*x*, 0}, *x* ^−^ = max⁡{−*x*, 0}, *h* > −min⁡{*c*_1_, *c*_2_}, and *b* > max⁡{*c*_1_, *c*_2_}. *h* > −min⁡{*c*_1_, *c*_2_} indicates that *h* could be positive or negative. When *h* > 0, it implies that there is need to take some costs to control extra inventory, while −min⁡{*c*_1_, *c*_2_} < *h* < 0 implies that we could obtain some incomes when selling extra stock. However the income could not make up the unit cost. The latter is shown in the newsboy problem. *b* > max⁡{*c*_1_, *c*_2_} means that the shortage cost is less than the unit cost.

When the holding and shortage cost function is piecewise linear, the result is simplified because both *x*_1_ and *x*_2_ are positive. Here we omit the proof. Then the optimal policy is as follows.

**Theorem 4 .**

When the holding and shortage cost function is piecewise linear, suppose that

(7) c1x1+EHx1−Dn=min⁡c1x+EHx−Dn,c2x2+EHx2−Dn=min⁡c2x+EHx−Dn,k2=c1min⁡{x1,Q}+E[H(min⁡{x1,Q}−Dn)]hhhh−c2min⁡{x2,Q}−E[H(min⁡{x2,Q}−Dn)].

Then the optimal ordering policy is to order min⁡{*x*_1_, *Q*} from supplier 1 if *K* ≥ *k*_2_ and min⁡{*x*_2_, *Q*} from supplier 2 if *K* ≤ *k*_2_. If *K* = *k*_2_, ordering from either supplier could be optimal.

Here we also omit the proof. And we will find that if the holding and shortage cost function is piecewise linear, no order will not be the optimal policy. Moreover, we only need to compare *K* and *k*_2_ to decide the ordering strategy.

## 4. Numerical Results

In order to explore the effects of the fixed setup cost *K* and the ordering capacity *Q* on the optimal control policy, we conduct several numerical experiments for a simple inventory problem with piecewise linear holding and shortage cost function. In the subsequent numerical experiments, we use the following basic settings: *c*_1_ = 5, *K* = 20, *c*_2_ = 3, and *Q* = 10. The demands could be 3, 6, 8, 10, and 15. And the corresponding possibilities are *p*_1_ = 0.1, *p*_2_ = 0.2, *p*_3_ = 0.3, *p*_4_ = 0.3, and *p*_1_ = 0.1. In the piecewise linear holding and shortage cost function, we assume that *h* = 2 and *b* = 7.

### 4.1. Effect of *K*

**Figure 1:**
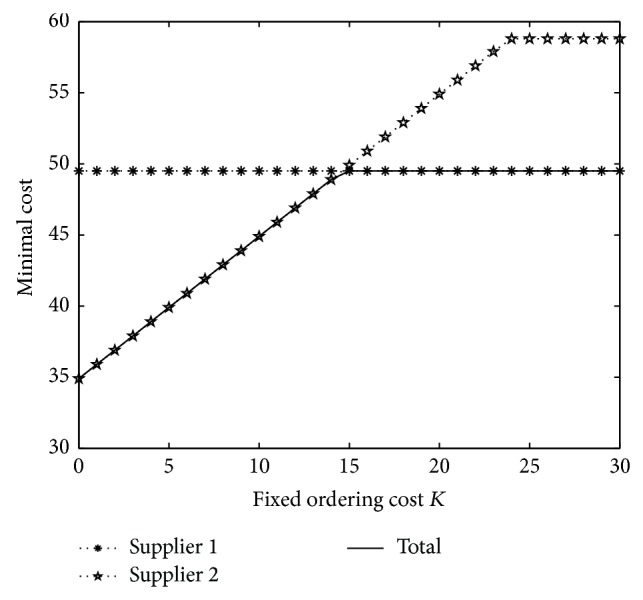
Minimal cost under different *K*.

Firstly, we consider the effect of fixed ordering cost *K*. Let *K* = 0,1,…, 30. The result is shown in Figure 1.

From Figure 1, it is obvious that fixed ordering cost *K* does not affect the optimal policy when ordering from supplier 1. However, when ordering from supplier 2, the minimal cost is increasing on *K*. If *K* is large enough, the optimal policy for ordering from supplier 2 will change from ordering some quantity to no order. As a whole, the best supplier will change from supplier 2 to supplier 1 when *K* is increasing.

### 4.2. Effect of *Q*

**Figure 2:**
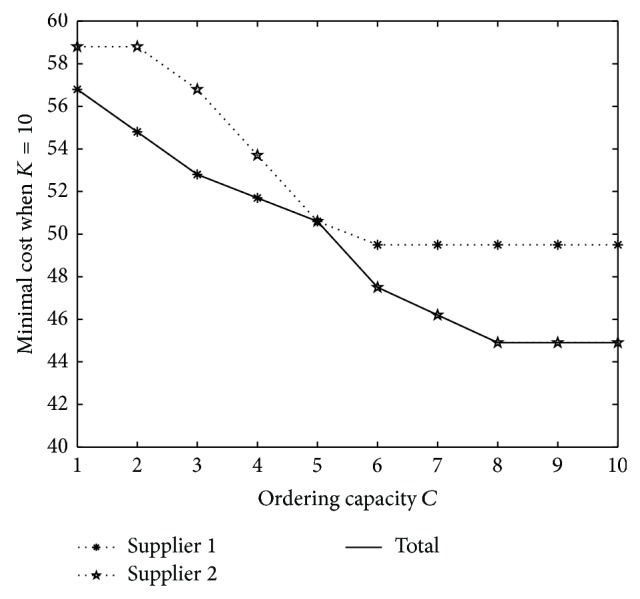
Minimal cost under different *Q* when *K* = 10.

**Figure 3:**
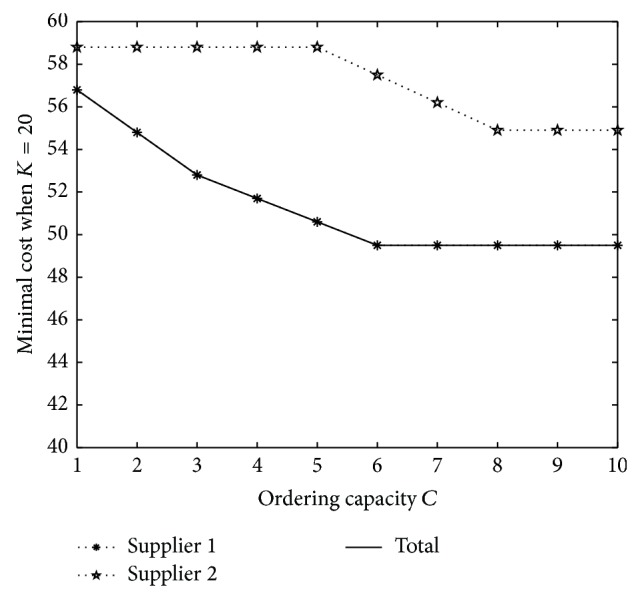
Minimal cost under different *Q* when *K* = 20.

Then we consider the effect of ordering capacity *Q*. Let *Q* = 1,…, 10. The result when *K* = 10 is shown in Figure 2 and the one when *K* = 20 is shown in Figure 3.

Then we will find that when ordering capacity *C* is small, the optimal ordering quantity is increasing and minimal cost is decreasing on *C*. However, when ordering capacity is large enough, the optimal policy is constant. Moreover, when *C* is increasing, the choice of supplier will change from supplier 1 to supplier 2 when *K* is small, while the choice will be always to order from supplier 1 when *K* is large.

## 5. Conclusion

In this paper, we consider a single-period inventory system with two suppliers. When we order from the first supplier, the ordering cost only includes variable cost. When we order from the other supplier, the ordering costs include a fixed cost and variable cost. And we could only order from one supplier. Moreover, there exists ordering capacity constraint. Then we establish the model and develop the optimal policy. We also conduct several numerical experiments to explore the effects of the fixed ordering cost *K* and the ordering capacity *Q* on the optimal control policy. In the future research, we will study the multiperiod inventory model.
